# Malignant Epithelioid Mesothelioma of the Tunica Vaginalis Testis Presenting as Hydrocele in a Kidney Transplant Recipient

**DOI:** 10.1155/2024/9227764

**Published:** 2024-01-10

**Authors:** Brett M. Behers, Christopher W. Guske, Benjamin J. Behers, Spencer B. Kortum, Isabella G. Bermingham, Christina L. Warner, Robert I. Carey

**Affiliations:** ^1^University of South Florida College of Medicine, Tampa, FL, USA; ^2^Florida State University College of Medicine, Tallahassee, FL, USA; ^3^SaraPath Diagnostics, Sarasota, FL, USA; ^4^Jellison Cancer Institute, Sarasota Memorial Hospital, Sarasota, FL, USA

## Abstract

Mesotheliomas of the tunica vaginalis testis are rare malignant tumors that can present as a scrotal mass or hydrocele. These tumors are typically aggressive with high rates of recurrence and metastasis. Suspected risk factors for malignant mesothelioma include asbestos exposure, chronic inflammation, trauma, and persistent hydrocele. We report the case of a malignant epithelioid mesothelioma of the tunica vaginalis testis that presented as a finding at hydrocelectomy and was ultimately treated with radical inguinal orchiectomy. This patient was on chronic immunosuppression therapy with tacrolimus and mycophenolate mofetil secondary to a kidney transplant but had none of the common risk factors for mesothelioma formation. To our knowledge, this is the first case describing a possible connection between chronic immunosuppression and mesothelioma of the tunica vaginalis. However, future studies are needed to investigate this association and discern whether this could have played a role in our patient or if his mesothelioma formation was coincidental.

## 1. Introduction

Mesotheliomas of the tunica vaginalis testis are rare malignant tumors, with less than 300 cases having been reported to date [1]. These tumors are typically aggressive with high rates of recurrence and metastasis. They are generally treated with radical resection, as radiation and chemotherapy have proven ineffective [1]. Potential risk factors for this malignancy include asbestos exposure, chronic inflammation, trauma, and persistent hydrocele [1, 2]. We present the case of a malignant epithelioid mesothelioma of the tunica vaginalis testis. The mesothelioma was diagnosed secondary to a recurrent hydrocele in a kidney transplant recipient (KTR) with a history of chronic immunosuppression and no other known risk factors.

## 2. Case Presentation

An 80-year-old male patient presented to our urology clinic complaining of right-sided scrotal swelling for the past six months. His medical history included a left-sided kidney transplant in 2012 for end-stage renal disease (ESRD) secondary to chronic bladder outlet obstruction. Following the transplant, the patient was put on long-term immunosuppressive therapy consisting of tacrolimus and mycophenolate mofetil.

His vital signs and urinalysis were unremarkable, aside from 3+ protein and 1+ blood in the urine. Ultrasound imaging determined the scrotal swelling to be a large, right-sided hydrocele with an otherwise unremarkable right testicle. Serous fluid was drained from the hydrocele in the office on three occasions without lasting relief. The patient then elected to proceed with a right hydrocelectomy.

**Figure 1:**
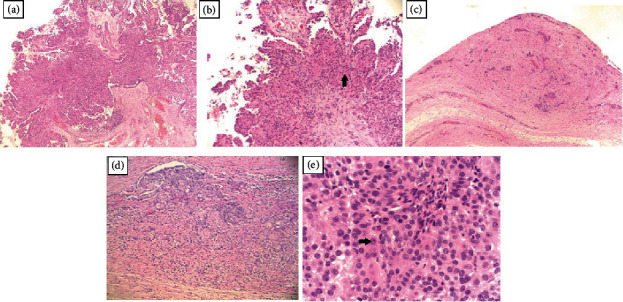
Histology pictures of the specimen on hematoxylin and eosin stain. (a) Papillary mesothelioma with epithelioid cells and thick fibrovascular cores (40x). (b) Papillary mesothelioma with epithelioid cells and focal stromal infiltration (black arrow, 100x). (c) Epithelioid mesothelioma with nested/infiltrative pattern (100x). (d) Epithelioid mesothelioma with nested/infiltrative pattern (200x). (e) Epithelioid mesothelioma with large nucleoli and atypical mitotic figure (black arrow, 400x).

**Figure 2:**
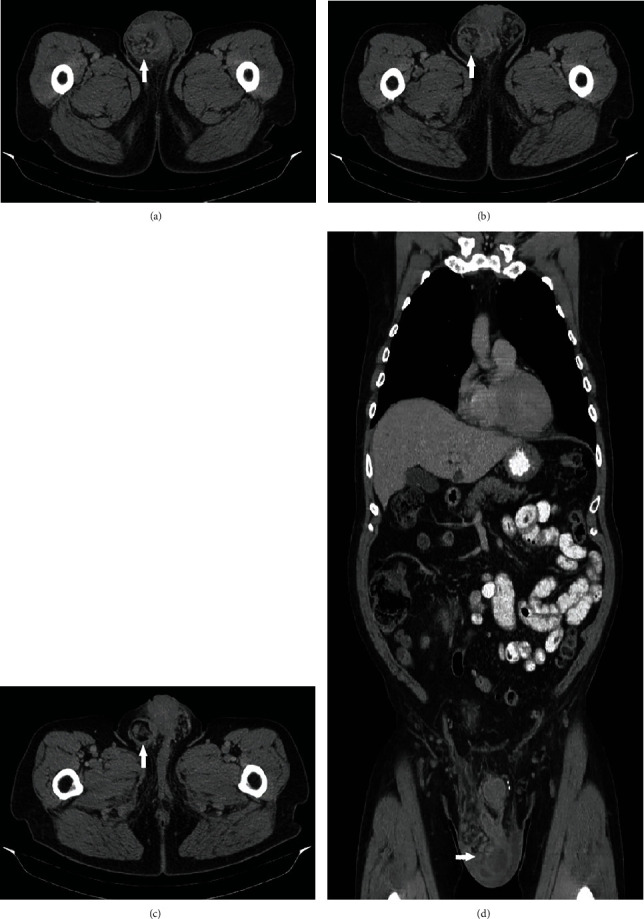
Computed tomography (CT) scan of the chest, abdomen, and pelvis noting a right-sided scrotal mass (white arrows) without signs of a primary tumor site or metastasis. Axial view (a-c) shows the scrotal mass (white arrow), and coronal view (d) shows the entire chest, abdomen, and pelvis.

The hydrocelectomy was uneventful; however, the patient's tunica vaginalis was found to be abnormally thickened. A specimen was sent to pathology, where hematoxylin and eosin staining revealed it to be an epithelioid malignant mesothelioma (Figure 1). The patient underwent computed tomography (CT) imaging of the chest, abdomen, and pelvis, which showed no evidence of metastasis. The decision to utilize CT imaging was made after the patient's insurance would not cover a positron emission tomography (PET) scan. A right-sided scrotal mass measuring 5.5 × 3.3 cm was noted, consistent with his primary mesothelioma of the tunica vaginalis testis (Figure 2). Of note, this mass was not present on a CT abdomen and pelvis one year prior.

**Figure 3:**
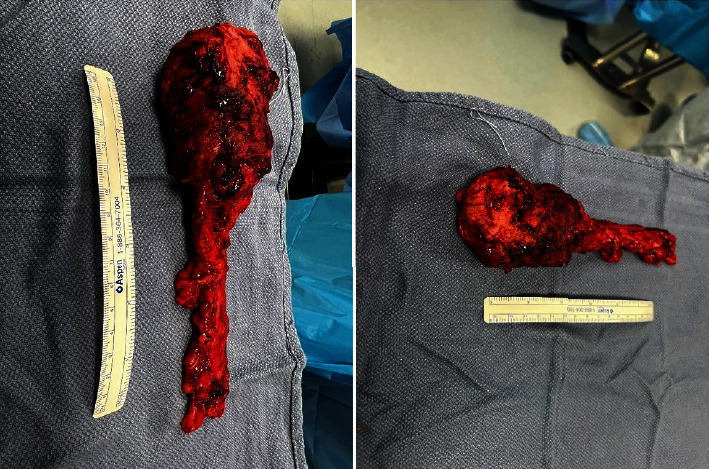
Gross images of the right-sided inguinal orchiectomy specimen.

Three weeks later, the patient returned to the operating room for a right-sided inguinal orchiectomy. Frozen sections were sent intraoperatively and noted to have clear margins. The specimen was sent off to pathology, where residual epithelioid malignant mesothelioma of the tunica vaginalis was noted in the right testicle, as well as residual hydrocele (Figure 3). There was no evidence of spread to the epididymis, rete testis, or seminiferous tubules.

The patient tolerated the procedure well and was seen four months later with a CT scan of the abdomen and pelvis, as well as a scrotal ultrasound, which showed no evidence of local recurrence and no evidence of metastases. No hydrocele was noted at that time, and his scrotal swelling had improved significantly. He did not receive any adjuvant chemotherapy or radiation therapy. Close follow-up with routine visits and CT scans are planned for surveillance.

## 3. Discussion

Mesothelial tissue is a mesoderm-derived layer of simple squamous epithelial cells that lines a number of body cavities, including the pleura, pericardium, peritoneum, and testicles. In the scrotum, this tissue forms a protective coating around the testes known as the tunica vaginalis. Mesotheliomas are tumors that can result from the malignant transformation of mesothelial cells. These transformations in the tunica vaginalis are exceedingly rare, accounting for just 0.3-1.4% of all malignant mesotheliomas, with less than 300 cases reported worldwide as of 2022 [1].

Testicular mesotheliomas often present as a painless scrotal mass or hydrocele. A hydrocele is an accumulation of fluid within the tunica vaginalis between its parietal and visceral layers, often due to obstruction of outflow [2]. Hydroceles can be treated conservatively with aspiration, but they have a high rate of recurrence. As a result, they often require a partial resection of the tunica vaginalis via hydrocelectomy. Resection remains the first-line treatment for mesotheliomas of the tunica vaginalis testis, as they are generally unresponsive to chemotherapy or radiation [3]. Radical orchiectomy appears superior to simple hydrocelectomy, as the rates of recurrence are 11% and 33%, respectively [1]. Following resection, serial CT scans are usually performed to monitor for recurrence.

The prognosis of mesotheliomas of the tunica vaginalis is typically poor. The mean survival time is 23 months, decreasing to 18 months when metastatic [1]. Metastases have been observed in approximately 33% of cases, most commonly in inguinal lymph nodes or the lungs [1]. These tumors are predominantly found in patients between the ages of 55 and 75. While their exact etiology is unknown, potential risk factors include asbestos exposure (reported in about a third of cases), chronic inflammation, persistent hydrocele, and testicular trauma [1, 2]. A thorough review of our patient's medical records and personal accounts failed to reveal any known risk factors, including the absence of smoking, testicular trauma, or any known exposure to asbestos. However, given the previous widespread use of asbestos, exposure cannot be ruled out.

However, an intriguing element of our patient's case is his history of a kidney transplant in 2012, followed by over a decade of immunosuppressive therapy consisting of tacrolimus and mycophenolate mofetil. In general, kidney transplant recipients (KTRs) have a two-to-threefold higher risk of developing de novo cancers compared to the general population [4], with a fourfold increase in mesotheliomas specifically [5]. This increased incidence of cancers is thought to be due to a combination of the effects of chronic kidney disease, posttransplant immunosuppression, and transplant patients' underlying comorbidities [4, 6].

In the absence of other risk factors, we speculate that the formation of our patient's mesothelioma may have been related to his status as a KTR or his use of immunosuppressive therapies. The association between immunosuppression and mesothelioma disease is not a novel claim. There have been documented cases of both mediastinal and pleural mesotheliomas in HIV patients without known asbestos exposure. In both cases, the investigators hypothesized that their patient's malignant mesotheliomas may have been associated with their HIV-induced immunosuppression [7, 8]. Of the most commonly observed malignancies following kidney transplants, several have been linked to oncogenic viruses, such as Kaposi sarcoma (linked to human herpesvirus 8) and lymphomas (linked to the Epstein-Barr virus) [4]. It is postulated that patients on chronic immunosuppressants are more susceptible to these viruses, leading to the increased incidence of their respective malignancies. Interestingly, simian virus 40 (SV40) is a virus with a growing body of evidence linking it to the formation of several malignancies, including mesotheliomas [9–11]. Moreover, SV40 infections have been known to reactivate during states of immunosuppression [10]. Akin et al. presented a similar case of a patient who developed a mesothelioma of the tunica vaginalis in the absence of risk factors and postulated that the presence of SV40 may have been a possible etiology [12]. However, the link between SV40 and mesothelioma formation remains controversial, and our patient did not undergo serological testing for SV40. Additionally, there is an inherently limited body of evidence at the intersection of SV40 infection, immunosuppression, and mesothelioma formation. This limits our ability to suggest it as a concrete etiology presently.

There is also the matter that immunosuppressant agents themselves can promote carcinogenesis. Our patient had been on a long-term regimen that included tacrolimus, which works through the inhibition of the intracellular enzyme calcineurin [4]. This ultimately inhibits the activation and proliferation of T-cells, which is its primary mechanism of action. However, tacrolimus is known to exert a range of other cellular effects that can promote carcinogenesis, including the upregulation of cytokines like TGF-beta and vascular endothelial growth factor, the inhibition of DNA repair, and the inhibition of apoptosis [4].

It must also be stated that our patient's mesothelioma may have been coincidental and unrelated to the above factors. Given the limited number of cases of testicular mesotheliomas in the literature, we hope that more investigation will be done into whether any causative relationship exists between the formation of mesotheliomas and KTR status, states of chronic immunosuppression, or the use of tacrolimus or other specific immunosuppressants.

Ultimately, we encourage providers monitoring transplant patients to keep testicular mesotheliomas in mind when encountering scrotal pathologies such as recurrent hydroceles. Given the rarity of this tumor and the uncertainty of its connection to posttransplant states, we cannot recommend any definitive changes to screening protocols. However, the threshold for ordering further testing is generally lower in transplant patients, which should be of some help, especially given that testicular tumors often carry the undesirable combination of being indolent in early stages and aggressive in their overall growth.

## 4. Conclusion

This report describes the discovery of a malignant epithelioid mesothelioma of the tunica vaginalis testis that was diagnosed incidentally during a hydrocelectomy and was ultimately treated with radical inguinal orchiectomy. Although rare, testicular mesotheliomas should be considered as a potential cause of scrotal masses or hydroceles. To our knowledge, this is the first case describing a mesothelioma of the tunica vaginalis in a patient on chronic immunosuppressive therapy following a kidney transplant. We believe that additional research is warranted to explore the potential relationships between mesothelioma formation, states of chronic immunosuppression, and kidney transplant status.

## Data Availability

The data used to support the findings of this study are available from the corresponding author upon request.

## Additional Points

*Significance*. This case report describes the discovery of a malignant epithelioid mesothelioma of the tunica vaginalis testis presenting as a finding at hydrocelectomy and ultimately treated with radical inguinal orchiectomy. Mesotheliomas of the tunica vaginalis are rare and typically aggressive tumors. Our patient lacked risk factors for mesothelioma formation but may have been predisposed due to his chronic immunosuppression following kidney transplant. If so, this would be the first case documenting mesothelioma of the tunica vaginalis from this phenomenon.

## References

[1] Iczkowski K. A. (2023). Malignant mesothelioma of tunica vaginalis testis: update for 2022. *Advances in Anatomic Pathology*.

[2] Dagur G., Gandhi J., Suh Y. (2017). Classifying hydroceles of the pelvis and groin: an overview of etiology, secondary complications, evaluation, and management. *Current Urology*.

[3] Nazemi A., Nassiri N., Pearce S., Daneshmand S. (2019). Testicular mesothelioma: an analysis of epidemiology, patient outcomes, and prognostic factors. *Urology*.

[4] Cheung C. Y., Tang S. C. W. (2019). An update on cancer after kidney transplantation. *Nephrology, Dialysis, Transplantation*.

[5] Piselli P., Serraino D., Segoloni G. P. (2013). Risk of *de novo* cancers after transplantation: results from a cohort of 7217 kidney transplant recipients, Italy 1997-2009. *European Journal of Cancer*.

[6] Lichtenberg S., Rahamimov R., Green H. (2017). The incidence of post-transplant cancer among kidney transplant recipients is associated with the level of tacrolimus exposure during the first year after transplantation. *European Journal of Clinical Pharmacology*.

[7] James C. O., Woods A. W., Arya P., Abuelgasim K. A., Heath L. T., Sitapati A. (2009). Mesothelioma in an HIV/AIDS patient without history of asbestos exposure: possible role for immunosuppression in mesothelioma: a case report. *Cases Journal*.

[8] Kordossis T., Kosmopoulou O., Zagoreos J. (1994). Malignant mesothelioma in an HIV-positive patient. *AIDS*.

[9] Martini F., Corallini A., Balatti V., Sabbioni S., Pancaldi C., Tognon M. (2007). Simian virus 40 in humans. *Infectious Agents and Cancer*.

[10] Carbone M., Gazdar A., Butel J. S. (2020). SV40 and human mesothelioma. *Translational Lung Cancer Research*.

[11] Jasani B., Gibbs A. (2012). Mesothelioma not associated with asbestos exposure. *Archives of Pathology & Laboratory Medicine*.

[12] Akin Y., Bassorgun I., Basara I., Yucel S. (2015). Malignant mesothelioma of tunica vaginalis: an extremely rare case presenting without risk factors. *Singapore Medical Journal*.

